# Efficacy and safety of percutaneous endoscopic cervical discectomy for cervical disc herniation: a systematic review and meta-analysis

**DOI:** 10.1186/s13018-022-03365-1

**Published:** 2022-12-01

**Authors:** Jinjie Zhang, Qiujun Zhou, Yan Yan, Jianlei Ren, Shenyu Wei, Haijia Zhu, Zhoufeng Song

**Affiliations:** 1 The First Affiliated Hospital of Zhejiang Chinese Medical University (Zhejiang Provincial Hospital of Chinese Medicine), 54 Post and Telegraph Road Uptown, Hangzhou, 310000 China; 2 Department of First Clinical Medical College, Zhejiang Chinese Medical University, Hangzhou, 310000 China; 3 The Third Affiliated Hospital of Beijing University of Chinese Medicine, No. 51 Anwai Xiaoguanjie, Chaoyang District, Beijing, 100029 China; 4 Department of Hepato-Pancreato-Biliary Surgery, The Second Affiliated Hospital, Zhejiang University School of Medicine, Zhejiang, 310000 China; 5 Hangzhou Children’s Hospital, Hangzhou, 310000 China

**Keywords:** Percutaneous endoscopic cervical discectomy, Cervical disc herniation, Efficacy and safety, Systematic review and meta-analysis

## Abstract

**Background:**

Since there are currently no systematic evidence-based medical data on the efficacy and safety of PECD, this meta-analysis pooled data from studies that reported the efficacy or safety of PECD for cervical disc herniation to examine the efficacy, recurrence and safety of using PECD to treat cervical disc herniation.

**Methods:**

We searched the PubMed, EMBASE and Cochrane Library databases for studies published from inception to July 2022. Nine nonrandomized controlled trials (non-RCTs) that reported the efficacy or safety of percutaneous endoscopic cervical discectomy for cervical disc herniation were included. We excluded duplicate publications, studies without full text, studies with incomplete information, studies that did not enable us to conduct data extraction, animal experiments and reviews. STATA 15.1 software was used to analyse the data.

**Results:**

The proportions of excellent and good treatment results after PECD for CDH were 39% (95% CI: 31–48%) and 47% (95% CI: 34–59%), respectively. The pooled results showed that the VAS scores at 1 week post-operatively (SMD = −2.55, 95% CI: − 3.25 to − 1.85) and at the last follow-up (SMD = − 4.30, 95% CI: − 5.61 to − 3.00) after PECD for cervical disc herniation were significantly lower than the pre-operative scores. The recurrence rate of neck pain and the incidence of adverse events after PECD for cervical disc herniation were 3% (95% CI: 1–6%) and 5% (95% CI: 2–9%), respectively. Additionally, pooled results show that the operative time (SMD = − 3.22, 95% CI: − 5.21 to − 1.43) and hospital stay (SMD = − 1.75, 95% CI: − 2.67to − 0.84) were all significantly lower for PECD than for ACDF. The pooled results also showed that the proportion of excellent treatment results was significantly higher for PECD than for ACDF (OR = 2.29, 95% CI: 1.06–4.96).

**Conclusion:**

PECD has a high success rate in the treatment of CHD and can relieve neck pain, and the recurrence rate and the incidence of adverse events are low. In addition, compared with ACDF, PECD has a higher rate of excellent outcomes and a lower operative time and hospital stay. PECD may be a better option for treating CHD.

**Supplementary Information:**

The online version contains supplementary material available at 10.1186/s13018-022-03365-1.

## Introduction

Cervical disc herniation (CDH) is a group of diseases based on cervical disc degeneration. The main symptom of CDH is arm pain in various locations depending on the level of intervertebral disc herniation and cervical nerve root compression [1]. Since the 1950s, anterior cervical discectomy and fusion (ACDF) has evolved as the gold standard surgical treatment for cervical disc herniation with the advantage of good fusion rates [2, 3]. With the increased use of surgery, an increasing number of surgery-related complications have been reported, such as adjacent segment degeneration, graft subsidence, reduced intervertebral space height, pseudoarthrosis and access-related complications [4, 5]. Therefore, a minimally invasive surgical option using the percutaneous endoscopic approach has been developed.

Minimally invasive or endoscopic discectomy techniques have evolved in lumbosacral, cervical and thoracic surgery since Hijikata et al. [6] and Kambin et al. [7] began applying percutaneous discectomy techniques. In the field of cervical endoscopy technology, researchers are actively seeking to explore further improvements. The percutaneous endoscopic cervical discectomy (PECD) prototype is fluoroscopically guided percutaneous cervical disc decompression without endoscopic visualization [8], which is increasingly widely used in the treatment of cervical disc herniation due to its advantages of fast recovery, less trauma and satisfactory clinical effect [9, 10]. Wang et al. investigated differences in these outcomes among women with different menopausal statuses. They found that there were no significant differences in the clinical or other related outcomes of long-term single or two consecutive levels of ACDF among women with different menopausal statuses. However, the early bony fusion rates and anterior FSU height loss rates were poorer in late postmenopausal patients than in premenopausal or early postmenopausal patients. Hence, it is important to protect late postmenopausal patients in the early post-operative period to guarantee solid bony fusion [11]. However, there are currently no systematic evidence-based medical data on the efficacy and safety of PECD. This meta-analysis included studies that reported the efficacy or safety of PECD for cervical disc herniation to determine the efficacy, recurrence and safety of PECD in the treatment of cervical disc herniation.

## Methods

### Literature inclusion and exclusion criteria

The inclusion criteria were as follows: the study type is a randomized controlled trial (RCT) or non-RCT study; the study reports the efficacy or safety of percutaneous endoscopic cervical discectomy for cervical disc herniation; and the language is limited to Chinese and English.

The exclusion criteria were as follows: repeated publication; studies without full text, incomplete information or inability to conduct data extraction; animal experiments; reviews and systematic reviews.

### Search strategy

In this meta-analysis, we searched the PubMed, EMBASE, and Cochrane Library databases from inception to July 2022. The search terms were as follows: “percutaneous endoscopic cervical discectomy”, “endoscopic cervical discectomy” and “cervical disc herniation”, “cervical disc herniations”.

### Literature screening and data extraction

Two researchers independently carried out the literature search, screening and information extraction. Disagreements were resolved via discussion or negotiation with a third person. The extracted data included the author, publication year of articles, country, study design, sample size, sex, age, duration (month), operation time (min), hospital stay (day), interventions, outcomes including excellent treatment rate, good treatment rate, VAS scores, neck pain recurrence rate and incidence of adverse events.

### Literature quality assessment

The quality of evidence for each study was assessed by two independent researchers using the Methodological Index for Nonrandomized Studies (MINORS) scale [12]. There are a total of 12 items; each item was scored on a scale from 0 to 2, and the maximum total score was 24. Studies with total scores ranging from 9 to 16 were classified as moderate quality, and those with scores ranging from 17 to 24 were classified as high quality.

### Data synthesis and statistical analysis

All data were analysed by STATA (version 15.1). I^2^ and Q tests were used to evaluate heterogeneity. If *P* ≥ 0.1 and *I*^2^ ≤ 50%, there was homogeneity between studies, and the fixed effects model was used for pooled analysis; if *P* < 0.1 or *I*^2^ > 50%, there was heterogeneity, and sensitivity analysis was used to find the source of heterogeneity. If the heterogeneity was still substantial, the random effects model was used, or the combination of results and descriptive analysis was omitted. A funnel plot and Egger’s test were used to assess publication bias.

## Results

### The results of the literature search

**Fig. 1:**
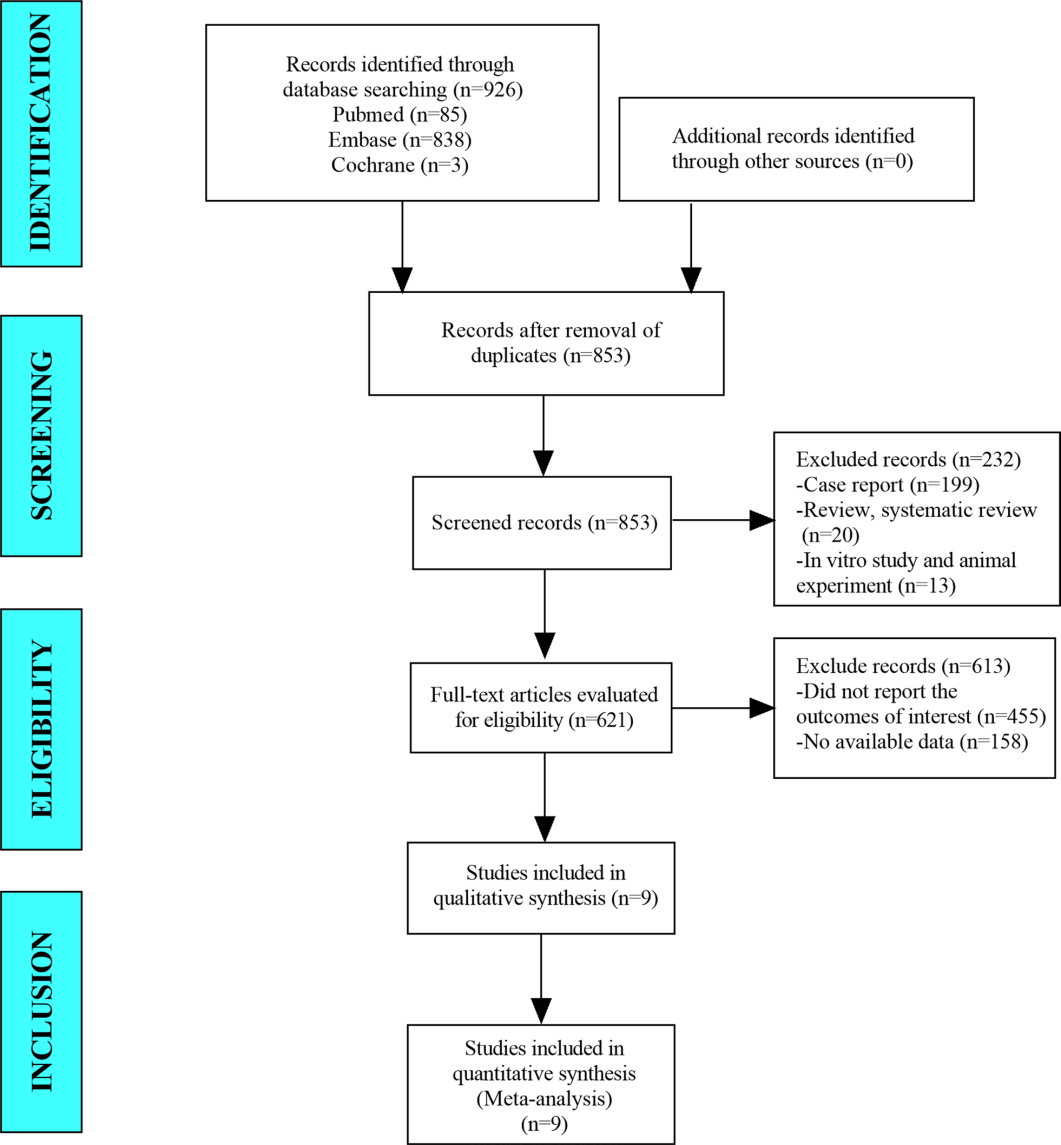
Flow diagram for the selection of studies

In this meta-analysis, a total of 926 studies were retrieved from the databases. After eliminating duplicate studies, 853 studies remained. After screening the titles and abstracts, 621 studies remained. Ultimately, 9 articles were included in the meta-analysis (Fig. 1).

### Baseline characteristics and quality assessment of the included studies

A total of 9 non-RCTs that reported the efficacy or safety of PECD for cervical disc herniation were included in this meta-analysis. The sample size of patients was a total of 390. Among them, there were 206 male patients and 184 female patients, and the ratio was close to 1. The average age of the patients was distributed in the range of 42.20–63.00, mainly in the middle-aged group. Four studies had patients from South Korea, and five studies had patients from China. The MINORS scores were all above 16 points, indicating that the included literature was of moderate or high quality (Table 1).

**Table 1:** Quality assessment of the baseline characteristics of the included studies

Author	Year	Country	Study design	Sample size	Gender (female/male)	Age	Duration (month)	Operation time (min)	Hospital stay (day)	Interventions	MINORS score
Ahn et al. [25]	2005a	Korea	Non-RCT	17	11/6	43.10	–	48.20 ± 13.75	–	PECD	16
Ahn et al. [26]	2005b	Korea	Non-RCT	36	17/19	43.10	–	–	–	PECD	16
Oh et al. [14]	2017	Korea	Non-RCT	100	35/65	46.90	4.80	–	–	PECD	17
Liao et al. [27]	2018	China	Non-RCT	22	13/9	48.80 ± 8.30	7.90 ± 3.20	69.70 ± 16.50	–	PECD	18
Shu et al. [28]	2019	China	Non-RCT	32	18/14	63.00 ± 10.50	–	56.00 ± 41.60	3.20 ± 1.30	PECD	17
Xiao et al. [29]	2019	China	Non-RCT	40	22/18	52.90 ± 14.30	11.20 ± 5.60	74.48 ± 7.08	3.86 ± 0.85	PECD	17
Jiang et al. [13]	2020	China	Non-RCT	36	13/23	59.89 ± 9.07	–	–	6.40 ± 0.74	PECD	16
Ahn et al. [15]	2020	Korea	Non-RCT	51	23/28	42.20	–	55.20 ± 18.03	2.18 ± 1.16	PECD	19
Ma et al. [16]	2021	China	Non-RCT	56	32/24	62.12 ± 2.36	6.22 ± 3.18	53.45 ± 4.12	4.34 ± 0.12	PECD	18

### Results of the meta-analysis

#### Treatment results

##### Excellent treatment results

**Fig. 2:**
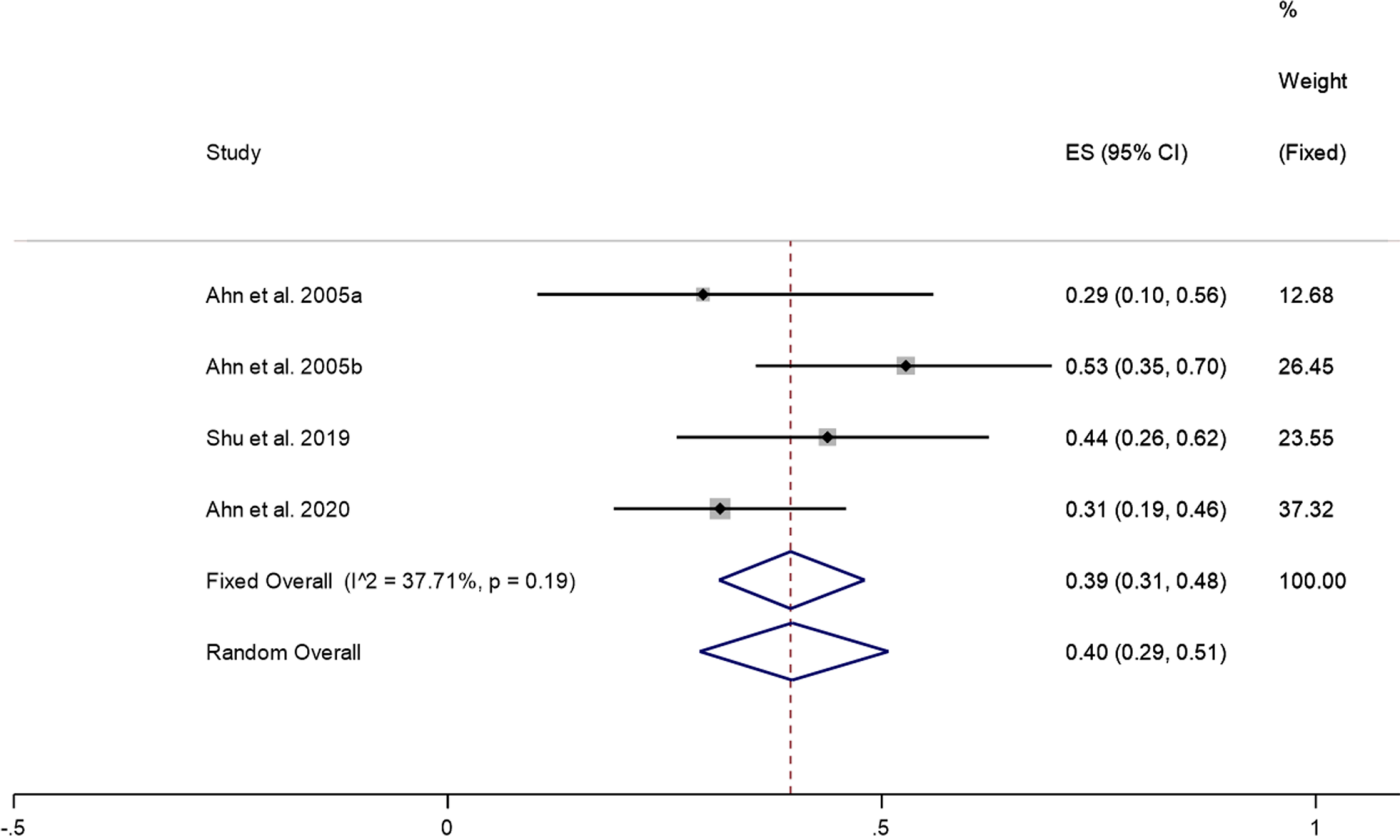
Excellent treatment results after PECD for cervical disc herniation

Five studies reported excellent treatment results after PECD for cervical disc herniation. Since there was significant heterogeneity (*I*^2^ = 90.72%, *P* = 0.00) (Additional file 1: Fig. S1), we conducted a sensitivity analysis and found that the study of Jiang et al. [13] had a substantial impact on the results. After removing their study, the heterogeneity test was performed again, and it was found that the heterogeneity was significantly reduced (*I*^2^ = 37.71%, *P* = 0.19). A fixed effects model was then used to perform meta-analysis. The pooled results showed that the prevalence of excellent treatment results after PECD for cervical disc herniation was 39% (95% CI: 31–48%) (Fig. 2).

##### Good treatment results

**Fig. 3:**
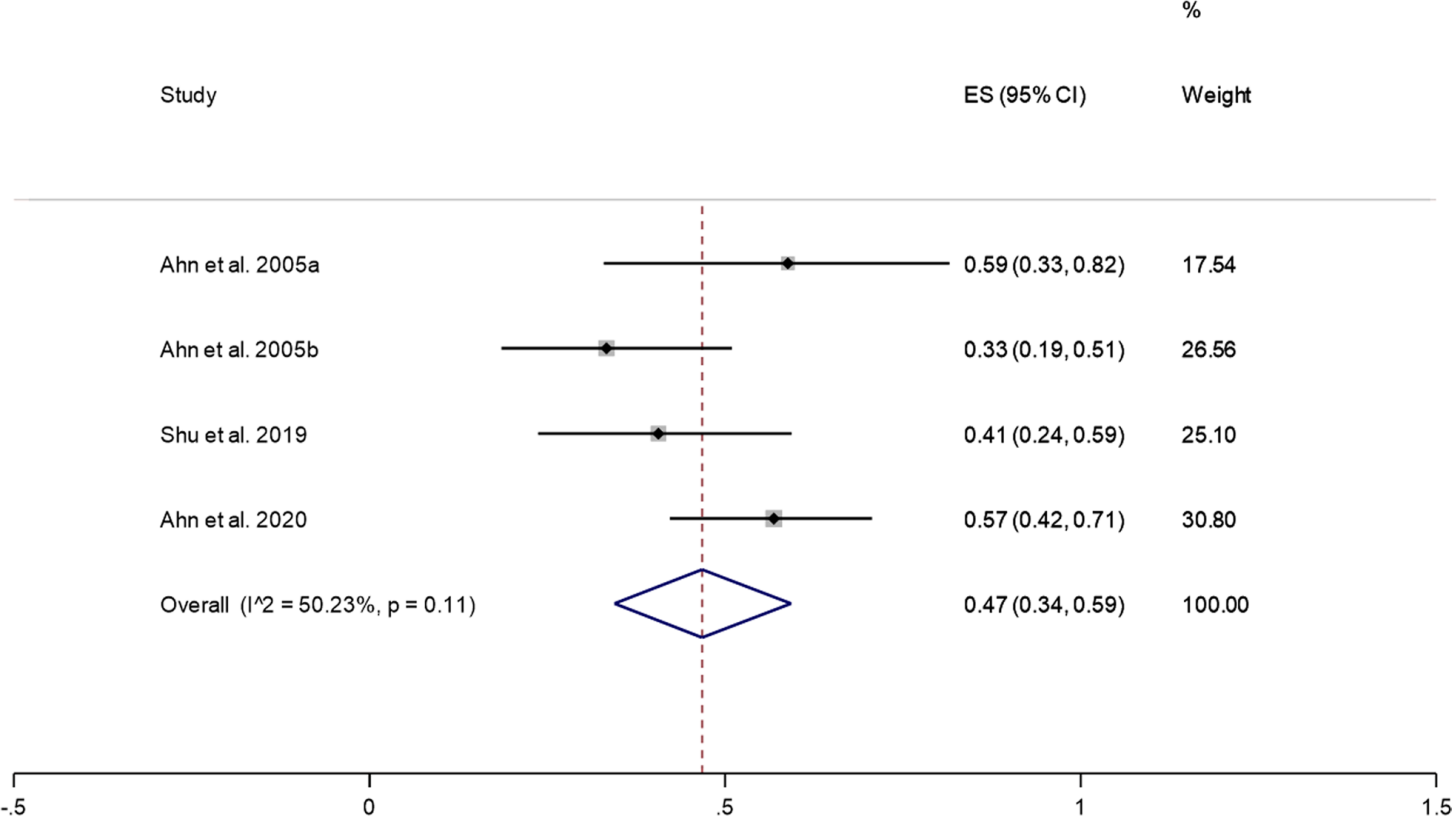
Good treatment results after PECD for cervical disc herniation

Five studies reported good treatment results after PECD for cervical disc herniation. Since there was significant heterogeneity (*I*^2^ = 82.06%, *P* = 0.00) (Additional file 1: Fig. S2), we conducted a sensitivity analysis and found that the study of Oh et al. [14] had a substantial impact on the results. After removing their study, the heterogeneity test was performed again, and it was found that the heterogeneity was significantly reduced (*I*^2^ = 50.23%, *P* = 0.11). A random effects model was used to perform meta-analysis. The pooled results showed that the prevalence of good treatment results after PECD for cervical disc herniation was 47% (95% CI: 34–59%) (Fig. 3).

#### VAS scores

The pooled results showed that VAS scores one week after PECD for cervical disc herniation were significantly lower than those pre-operatively (SMD =  − 2.55, 95% CI: − 3.25 to − 1.85, *P* = 0.000; I^2^ = 65.6%, *P* = 0.088; 2 studies).

**Fig. 4:**
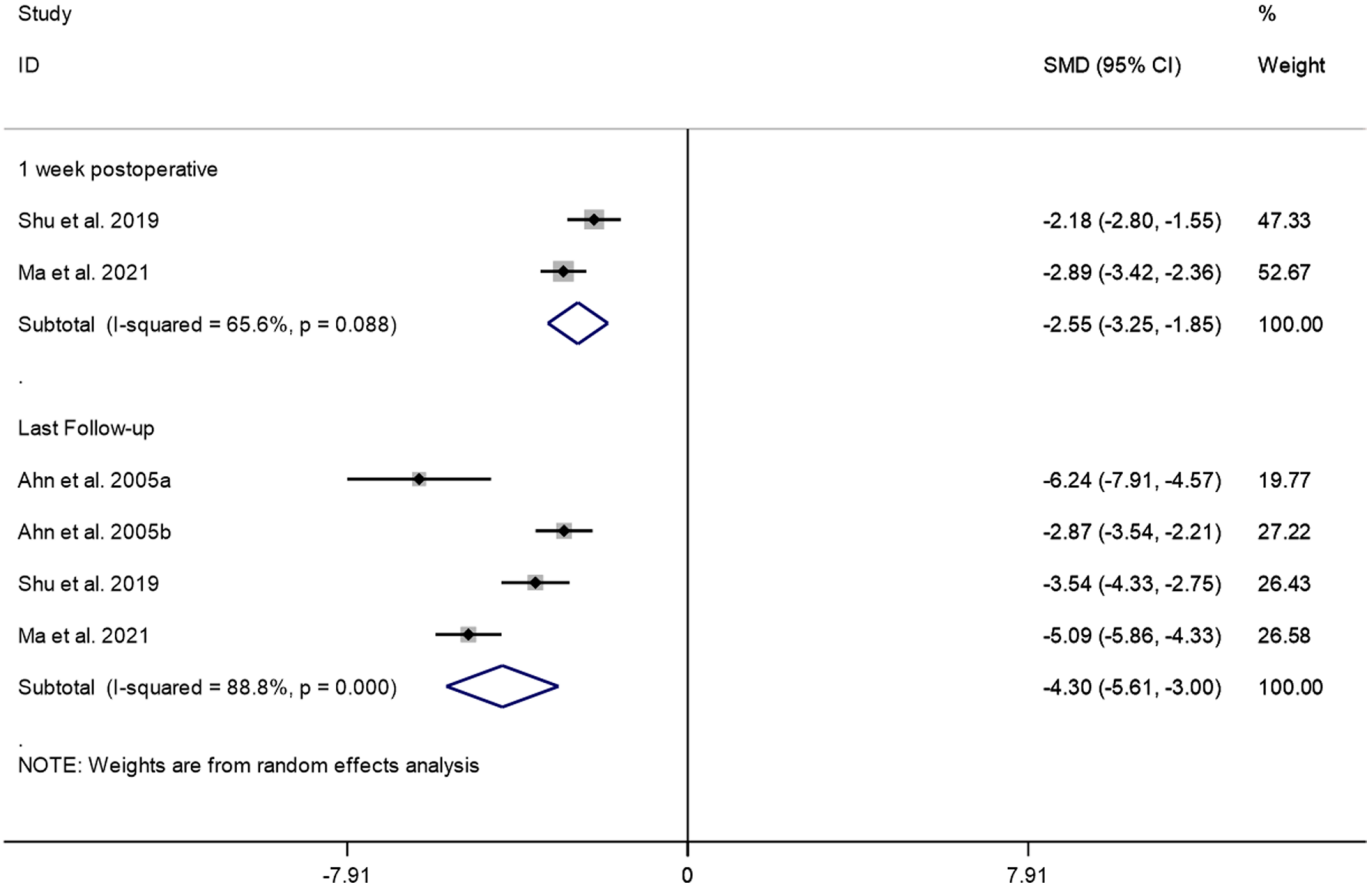
VAS score at 1 week and at the last follow-up after PECD for cervical disc herniation

In addition, 5 studies reported VAS scores at the last follow-up after PECD for cervical disc herniation. Since there was significant heterogeneity (*I*^2^ = 93.8%, *P* = 0.000) (Additional file 1: Fig. S3), we conducted a sensitivity analysis and found that the study of Ahn et al. [15]. Ahn et al. [15] have a substantial impact on the results. After removing their study, the heterogeneity test was performed again, and it was found that the heterogeneity was reduced (*I*^2^ = 88.8%, *P* = 0.000). A random effects model was used to perform meta-analysis. The pooled results showed that VAS scores at the last follow-up after PECD for cervical disc herniation were significantly lower than the pre-operative scores (SMD =  − 4.30, 95% CI: − 5.61 to − 3.00; *P* = 0000) (Fig. 4).

#### Neck pain recurrence rate

**Fig. 5:**
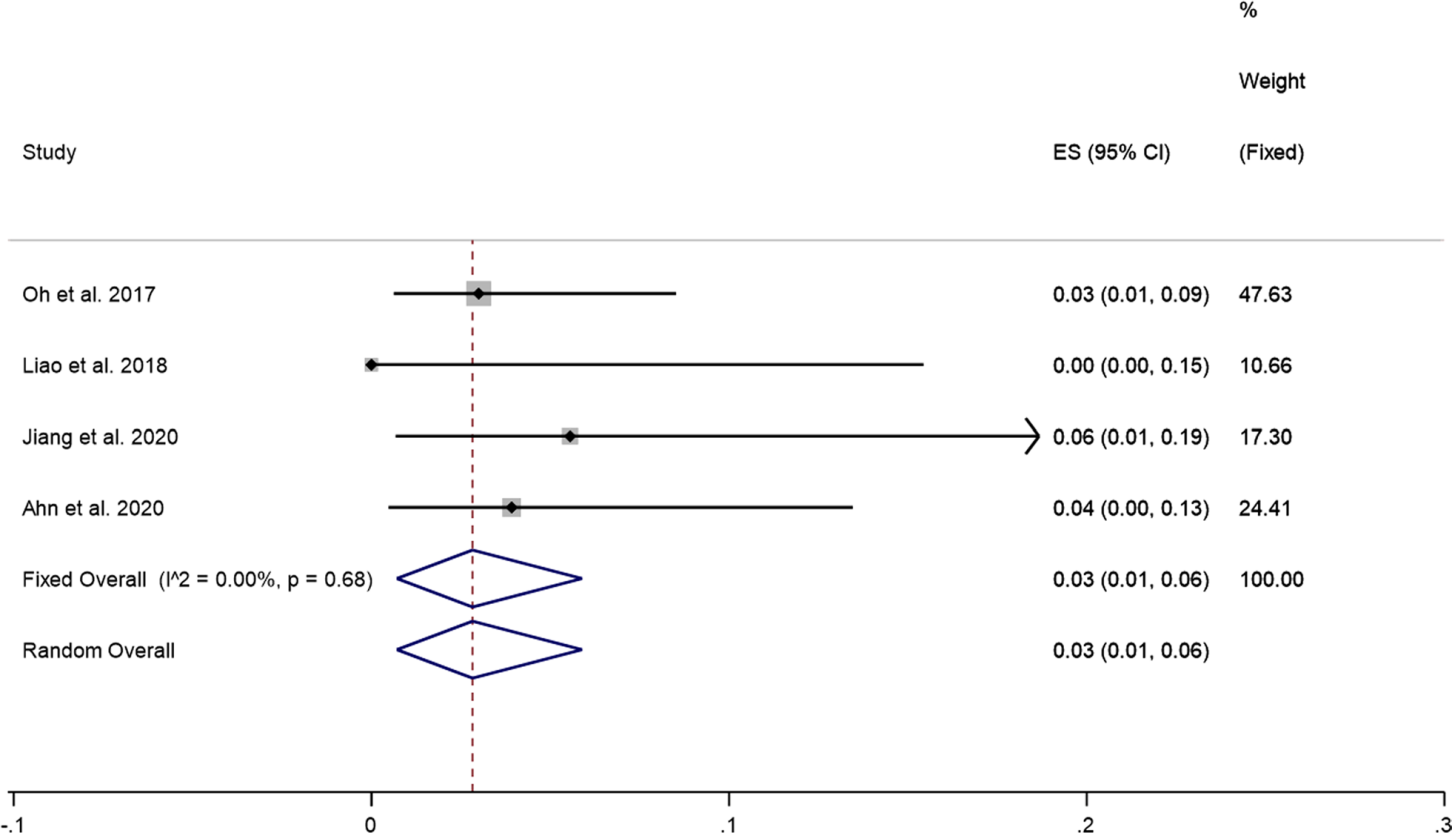
Prevalence of neck pain recurrence after PECD for cervical disc herniation

Four studies reported the neck pain recurrence rate after PECD for cervical disc herniation. Since there was no significant heterogeneity (*I*^2^ = 0.00%, *P* = 0.68), a fixed effects model was used to perform meta-analysis. The pooled results showed that the prevalence of neck pain recurrence after PECD for cervical disc herniation was 3% (95% CI: 1–6%) (Fig. 5).

#### Incidence of adverse events

**Fig. 6:**
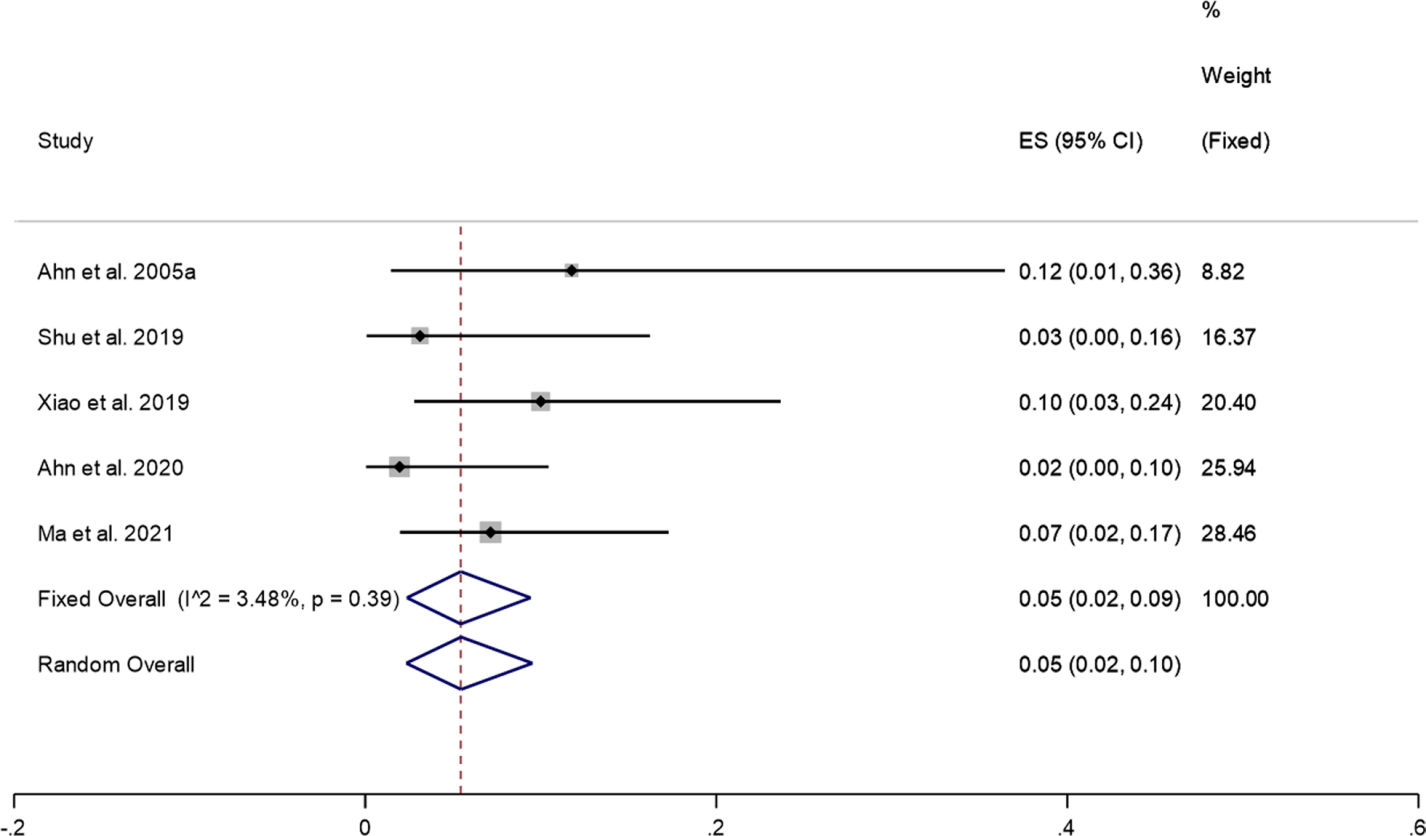
Prevalence of adverse events after PECD for cervical disc herniation

Five studies reported the incidence of adverse events after PECD for cervical disc herniation. Since there was no significant heterogeneity (*I*^2^ = 3.48%, *P* = 0.39), a fixed effects model was used to perform meta-analysis. The pooled results showed that the prevalence of adverse events after PECD for cervical disc herniation was 5% (95% CI: 2–9%) (Fig. 6).

#### Comparison of the efficacy and safety of PECD and anterior cervical discectomy and fusion (ACDF)

##### Operation time

**Fig. 7:**
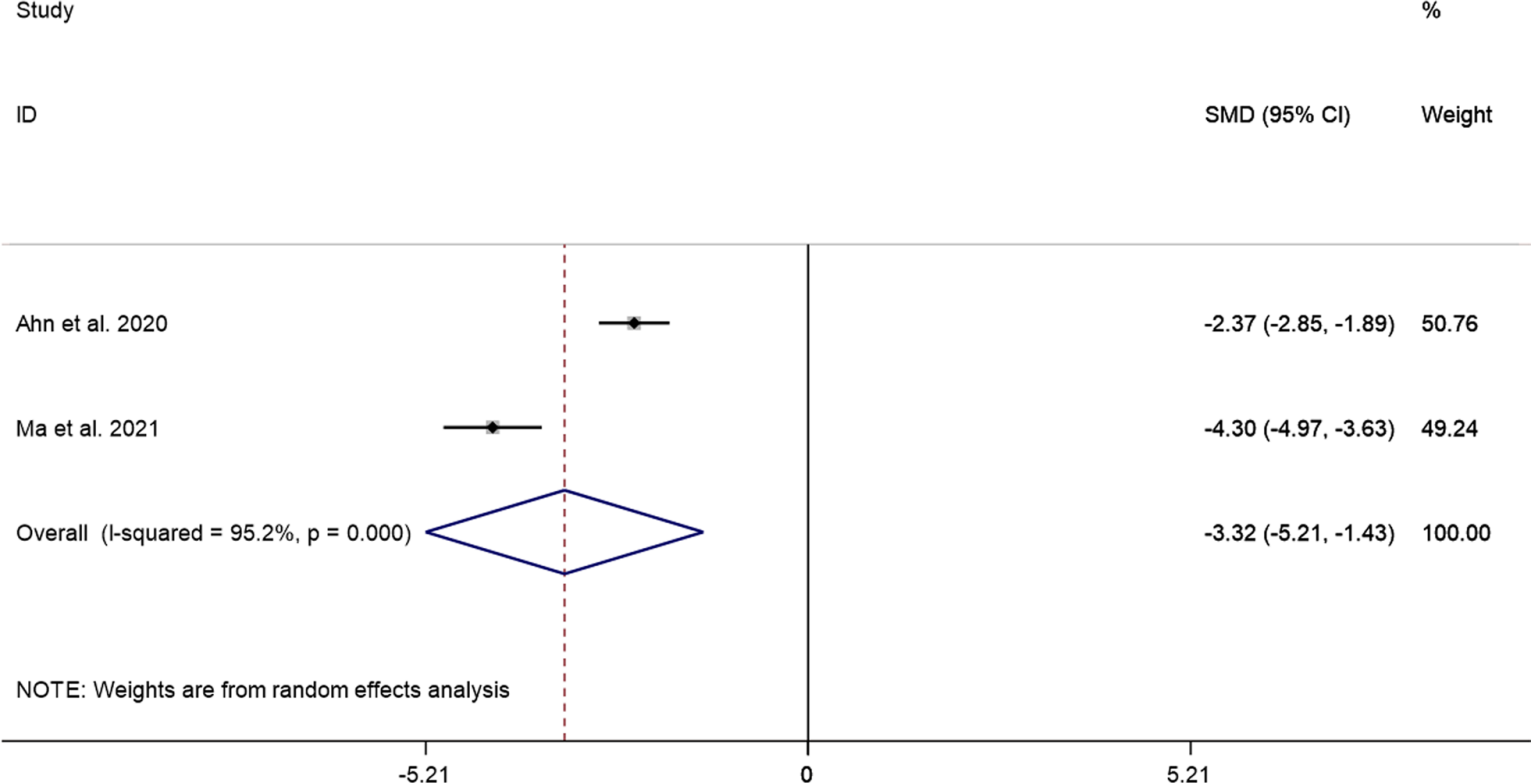
Comparison of operation time between the PECD group and the ACDF group

The pooled results showed that the operative time of PECD was significantly lower than that of ACDF (SMD =  − 3.22, 95% CI: − 5.21 to − 1.43, *P* = 0.001; *I*^2^ = 95.2%, *P* = 0.000; 2 studies) (Fig. 7).

##### Hospital stay

**Fig. 8:**
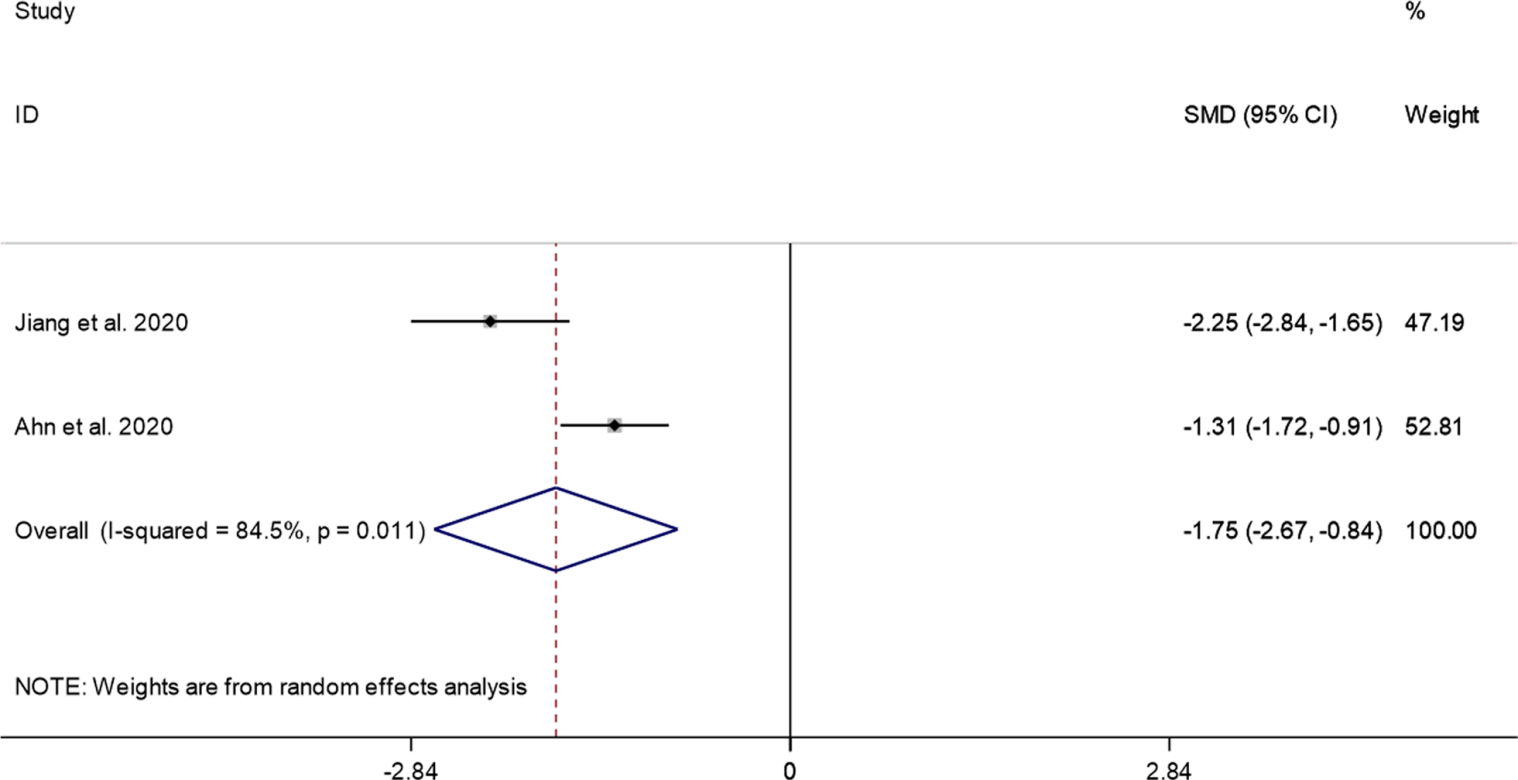
Comparison of hospital stay between the PECD group and the ACDF group

Three studies reported the hospital stay of the PECD group and the ACDF group. Since there was significant heterogeneity (*I*^2^ = 94.3%, *P* = 0.000) (Additional file 1: Fig. S4), we conducted a sensitivity analysis and found that the study of Ma et al. [16] had a substantial impact on the results. After removing their study, the heterogeneity test was performed again, and it was found that the heterogeneity was reduced (*I*^2^ = 84.5%, *P* = 0.000). A random effects model was used to perform meta-analysis. The pooled results showed that the hospital stay of the PECD group was significantly shorter than that of the ACDF group (SMD =  − 1.75, 95% CI: − 2.67 to − 0.84; *P* = 0000) (Fig. 8).

##### Excellent treatment results

**Fig. 9:**
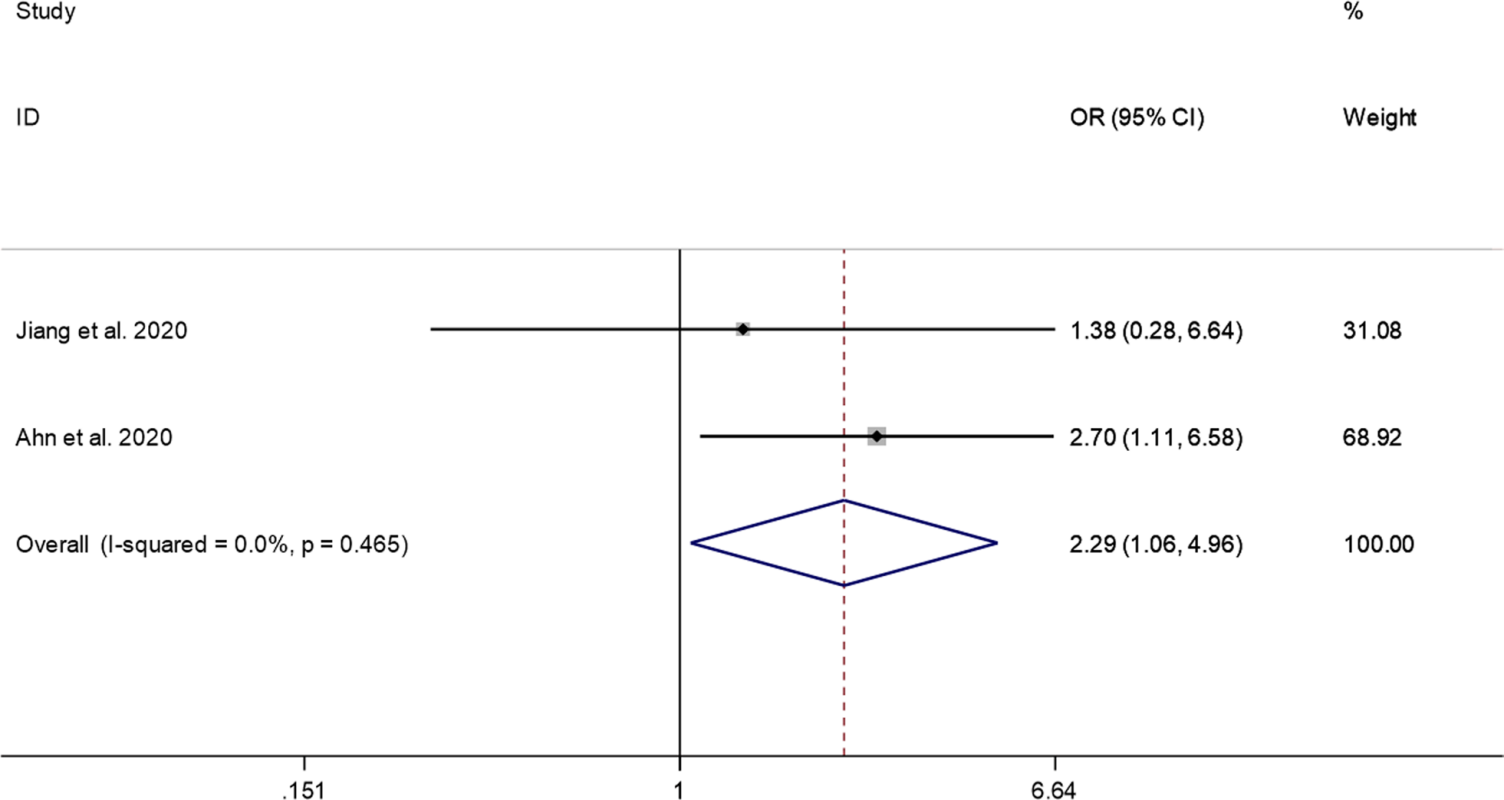
Comparison of excellent treatment results between the PECD group and the ACDF group

The pooled results showed that the prevalence of excellent treatment results was significantly higher in the PECD group than in the ACDF group (OR = 2.29, 95% CI: 1.06–4.96, *P* = 0.036; *I*^2^ = 0.0%, *P* = 0.465; 2 studies) (Fig. 9).

##### VAS scores at the last follow-up

**Fig. 10:**
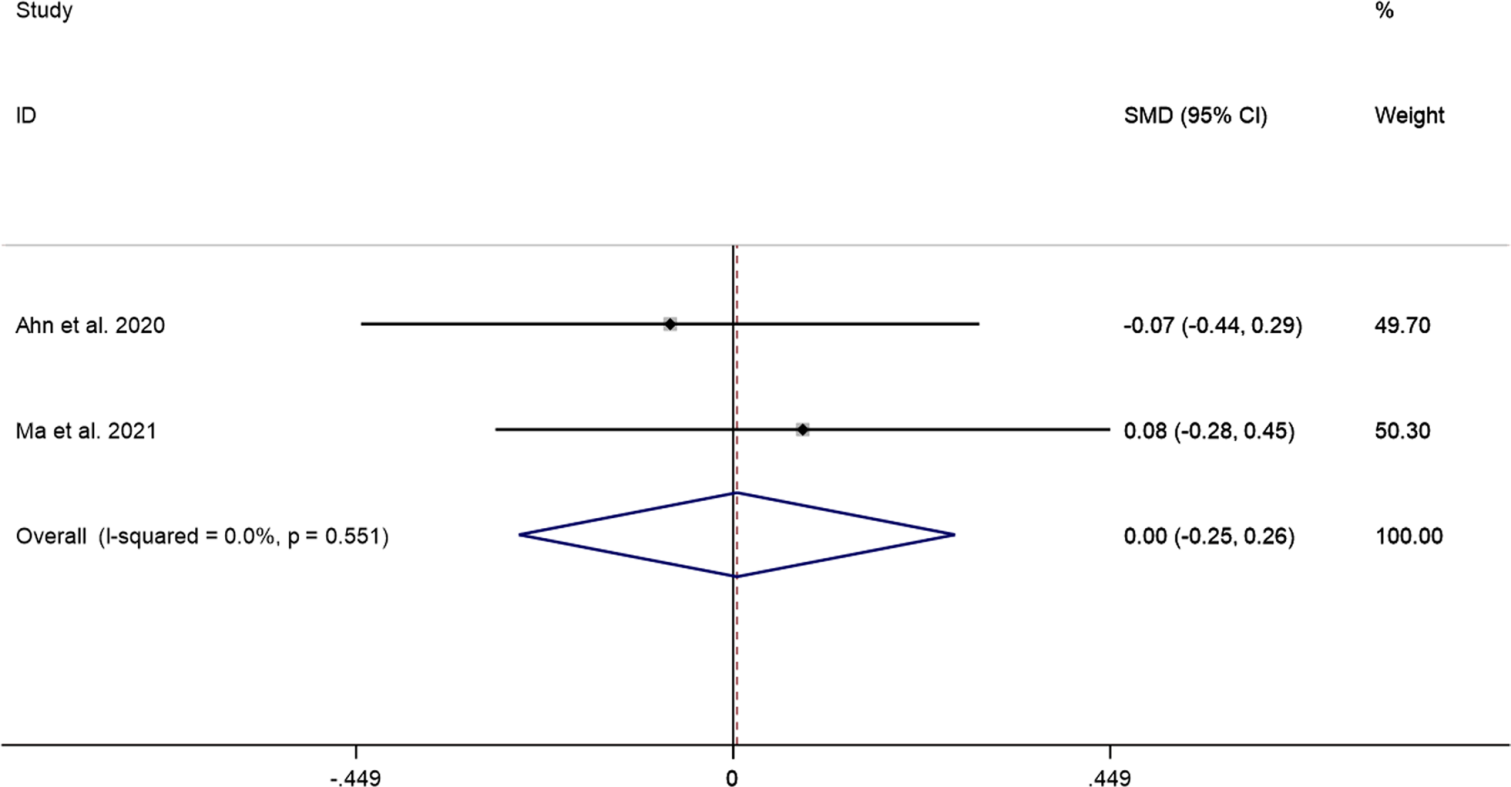
Comparison of VAS scores at the last follow-up between the PECD group and the ACDF group

The pooled results showed that the difference in VAS scores at the last follow-up between the PECD group and the ACDF group was not statistically significant (SMD = 0.00, 95% CI: − 0.25 to 0.26, *P* = 0.973; *I*^2^ = 0.0%, *P* = 0.551; 2 studies) (Fig. 10).

##### Neck pain recurrence rate

**Fig. 11:**
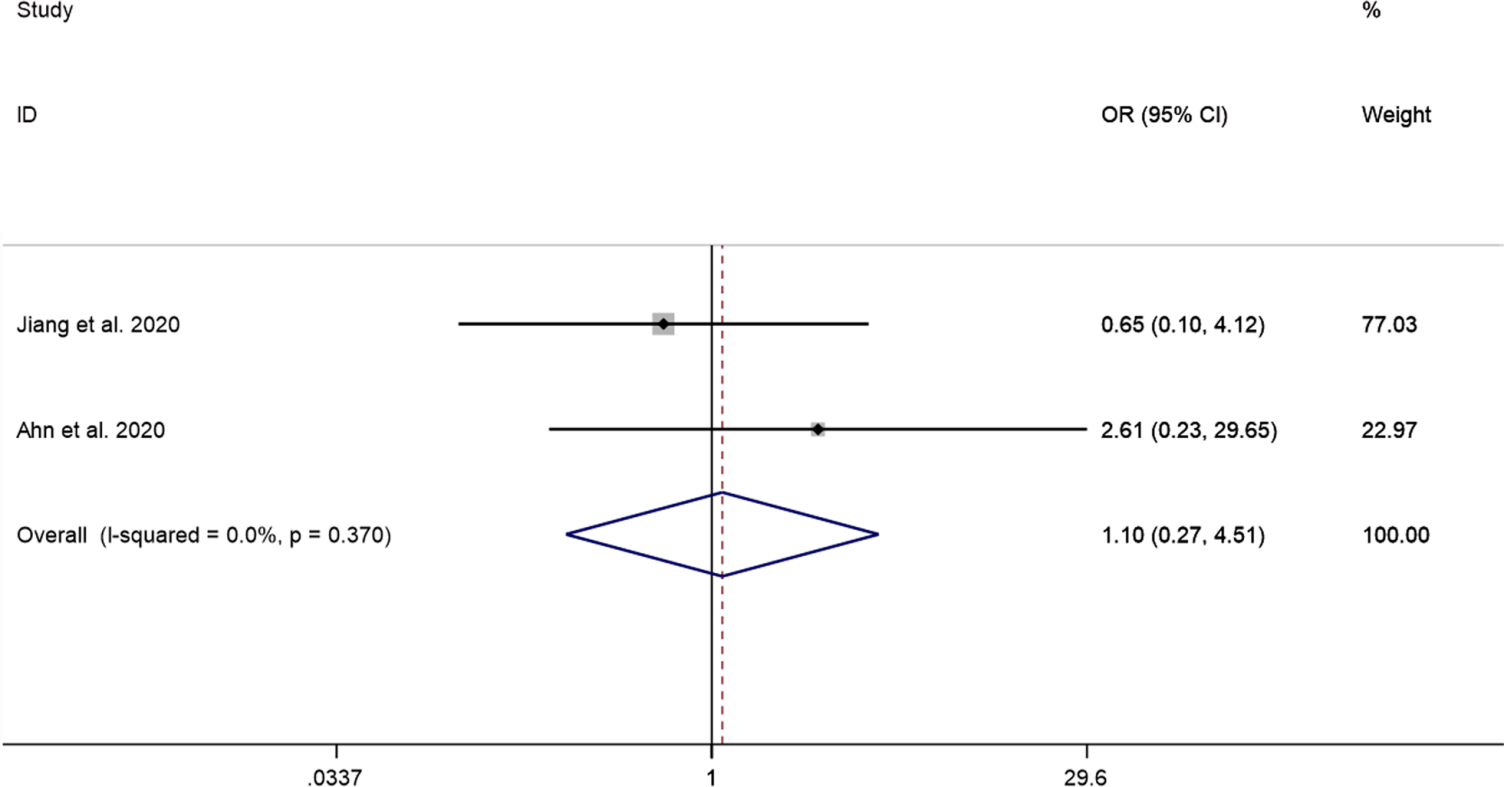
Comparison of the prevalence of neck pain recurrence rates between the PECD group and the ACDF group

The pooled results showed that the difference in the neck pain recurrence rate between the PECD group and the ACDF group was not statistically significant (OR = 1.10, 95% CI: 0.27–4.51, *P* = 0.896; *I*^2^ = 0.0%, *P* = 0.370; 2 studies) (Fig. 11).

##### Incidence of adverse events

**Fig. 12:**
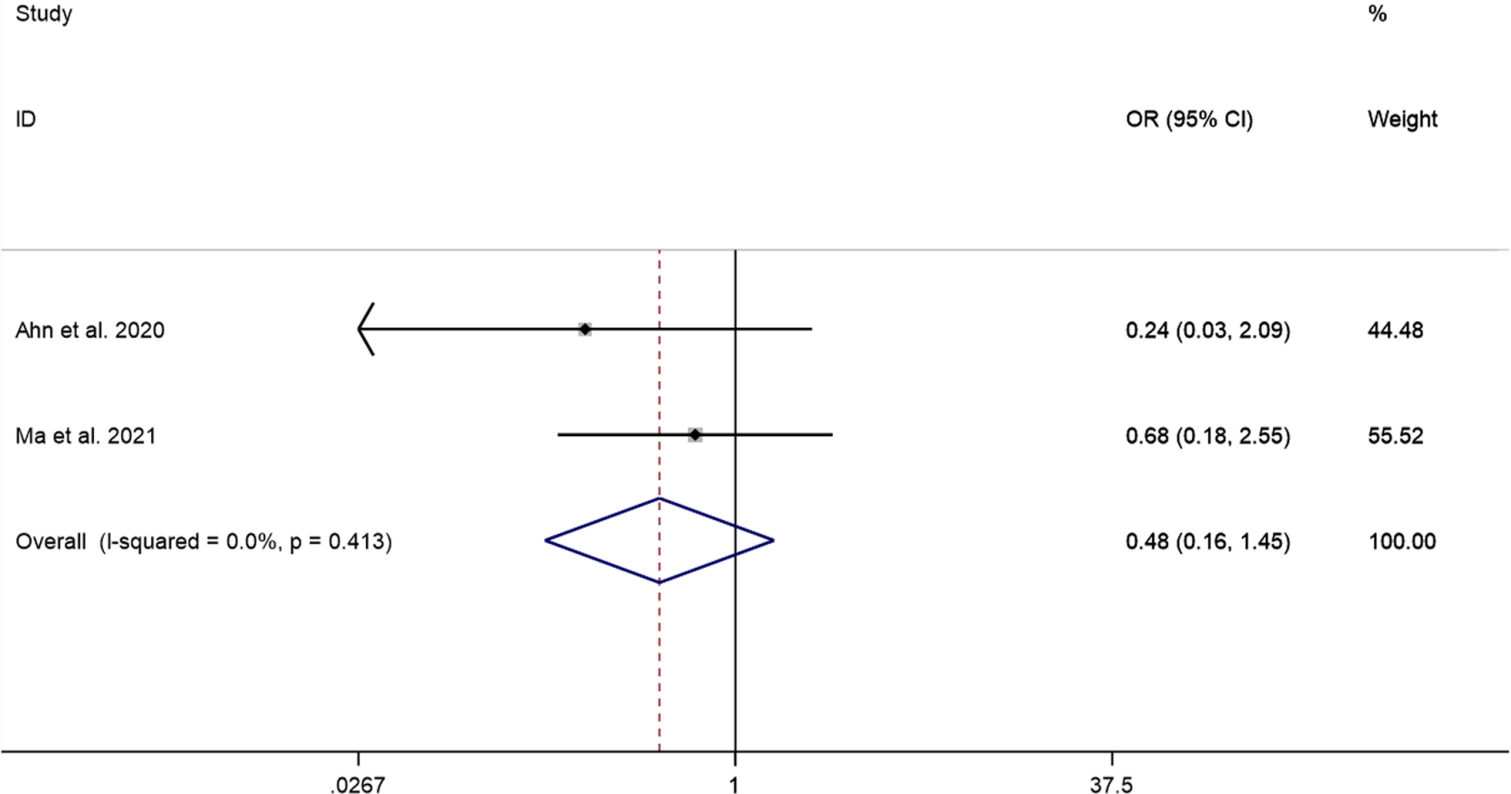
Comparison of the prevalence of adverse events between the PECD group and the ACDF group

The pooled results showed that the difference in the incidence of adverse events between the PECD group and the ACDF group was not statistically significant (OR = 0.48, 95% CI: 0.16–1.45, *P* = 0.194; I^2^ = 0.0%, *P* = 0.413; 2 studies) (Fig. 12).

### Sensitivity analysis

We conducted a sensitivity analysis by eliminating each included study on at a time and performing a summary analysis of the remaining studies. The results of the sensitivity analysis are shown in Additional file 1: Figs. S5–S8.

### Publication bias

**Fig. 13:**
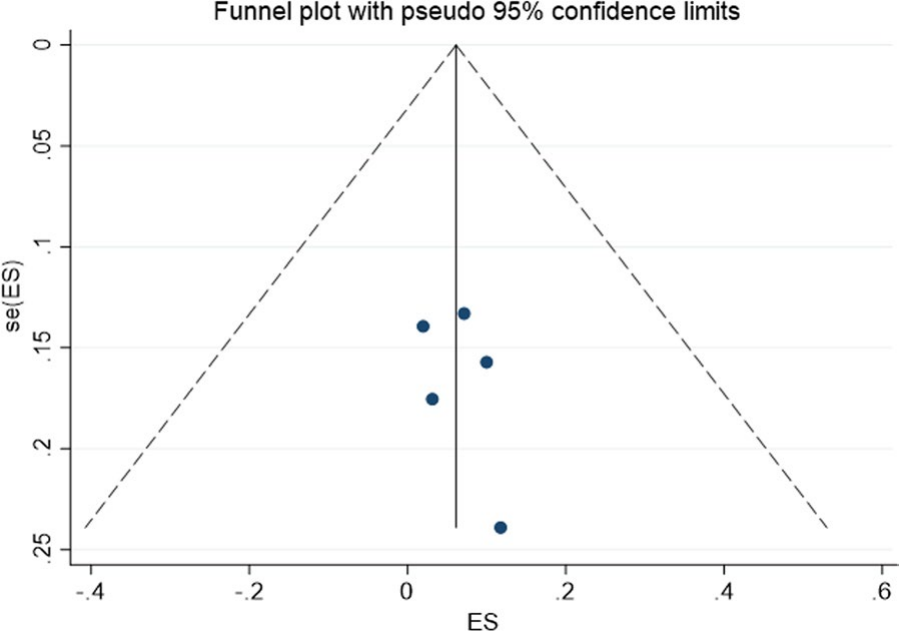
Funnel plot for assessing publication bias

The funnel plot drawn in this study is shown in Fig. 13. The funnel plot was basically symmetrical, and the P value of Egger's test was 0.478, indicating that there was no obvious publication bias in this study.

## Discussion

In the era of rapid information development, people are dependent on computers and mobile phones for life, entertainment, and work. Currently, the onset of CDH is seriously increasing in the younger population. Clinical data suggest that the imbalance between the internal and external cervical spine may be an important cause of CDH. Therefore, exploring effective treatment and prevention strategies to restore the dynamic and static balance of the cervical spine is the key to the treatment of CDH [17]. PECD has been regarded as an effective treatment modality in selected cases [18–20]. However, there is no systematic conclusion on the efficacy and safety of PECD in the treatment of CDH. This study included 9 reports reporting the efficacy or safety of PECD for CDH, including 390 patients, and analysed treatment outcomes, pain, recurrence and incidence of adverse events. Additionally, the advantages and disadvantages of PECD and ACDF on these indicators were compared.

First, the results pooled in this study showed that the prevalence of excellent and good treatment results after PECD for CDH was 39% and 47%, respectively. The combined prevalence of excellent and good treatment results reached 86%, which indicates that the success rate of PEDF in treating CDH is close to 90%. A 25-year follow-up study conducted by Burkhardt et al. reported a clinical success rate of 86.1% when using ACDF to treat CDH [21], which indicates that the success rate of PECD in the treatment of CDH is not inferior to that of ACDF. This study further compared the advantages and disadvantages of PECD and ACDF in excellent treatment results. Notably, pooled results show that the prevalence of excellent treatment results was significantly higher for the PECD group than for the ACDF group (OR = 2.29, 95% CI: 1.06–4.96). This finding shows that although the success rates of the two are similar, PECD can further improve the excellent rate of surgery, thereby improving patient satisfaction. This may be due to the characteristics of the endoscope itself. The percutaneous cervical approach with only a thin working channel endoscope of 3–4 mm diameter can minimize normal tissue trauma. Therefore, there is a reduction in scarring and faster rehabilitation when compared with open surgery [22].

Furthermore, the results pooled in this study showed that VAS scores after PECD for cervical disc herniation were significantly lower than those pre-operatively. This shows that PECD is effective in relieving neck pain, which can alleviate neurological symptoms by preserving some of the healthy discs rather than removing them altogether. Although pooled results show that the difference in VAS scores at the last follow-up between the PECD group and the ACDF group was not statistically significant, the way PECD relieves pain can delay the degeneration of adjacent segments [23]. Ma et al. [16] reported that 3 patients (3.3%) in the ACDF group developed adjacent segment disease within two years after surgery, while none developed adjacent segment disease in the PECD group. The pooled results also showed that the neck pain recurrence rate after PECD for cervical disc herniation was only 3%, which further suggests that the long-term efficacy of PECD is stable.

This study also focused on the occurrence of adverse events in PECD. PECD is punctured to the site of disc herniation through the interstitial space between the internal cervical sheath and the vascular sheath. During puncture, there is a risk of carotid artery, thyroid, superior larynx, recurrent larynx and oesophageal damage [24]. Notably, pooled results showed that the prevalence of adverse events after PECD for cervical disc herniation was 5% (95% CI: 2–9%). The pooled results suggest that the incidence of post-operative adverse events in PECD is not high. Although the results of this study suggest that the difference in the prevalence of adverse events between the PECD group and the ACDF group was not statistically significant, the objectivity of the results is challenged by the small number of studies included.

In addition to the analysis of the above indicators, this study also compared the perioperative differences between PECD and ACDF. The pooled results showed that the operative time and hospital days were significantly lower for the PECD group than for the ACDF group. This suggests that PECD can help improve surgical satisfaction and reduce medical costs for patients.

This study also has some limitations. First, there was significant heterogeneity in several indicators in this study. However, due to the lack of included literature, it is impossible to explore the source of heterogeneity. Second, due to the lack of comparative studies on the efficacy and safety of PECD and ACDF, the reliability of the results of this study is challenged. In the future, more large-scale randomized controlled trials are needed to verify the results of this study.

## Conclusion

PECD has a high success rate in the treatment of CHD and can relieve neck pain, and the recurrence rate and the incidence of adverse events are low. In addition, compared with ACDF, PECD has a higher rate of excellent outcomes and a lower operative time and hospital stay. PECD may be a better option for treating CHD.

## Supplementary Information

**Additional file 1: Figure S1**. Excellent treatment results after PECD for cervical disc herniation. **Figure S2**. Good treatment results after PECD for cervical disc herniation. **Figure S3**. VAS scores at the last follow-up after PECD for cervical disc herniation. **Figure S4**. Comparison of hospital stay between the PECD group and the ACDF group. **Figure S5**. Sensitivity analysis of excellent treatment results after PECD for cervical disc herniation. **Figure S6**. Sensitivity analysis of good treatment results after PECD for cervical disc herniation. **Figure S7**. Sensitivity analysis of VAS scores at the last follow-up after PECD for cervical disc herniation. **Figure S8**. Sensitivity analysis of the comparison of hospital stay between the PECD group and the ACDF group.

## Data Availability

The datasets are available from the corresponding author on reasonable request.
