# Supplementary material for: Efficacy and safety of percutaneous endoscopic cervical discectomy for cervical disc herniation: a systematic review and meta-analysis
Source: J Orthop Surg Res. 2022 Dec 1;17:519. doi: 10.1186/s13018-022-03365-1 (PMC9714009; doi:10.1186/s13018-022-03365-1)
Supplement: Supplementary file 1 — Additional file 1: Figure S1. Excellent treatment results after PECD for cervical disc herniation. Figure S2. Good treatment results after PECD for cervical disc herniation. Figure S3. VAS scores at the last follow-up after PECD for cervical disc herniation. Figure S4. Comparison of hospital stay between the PECD group and the ACDF group. Figure S5. Sensitivity analysis of excellent treatment results after PECD for cervical disc herniation. Figure S6. Sensitivity analysis of good treatment results after PECD for cervical disc herniation. Figure S7. Sensitivity analysis of VAS scores at the last follow-up after PECD for cervical disc herniation. Figure S8. Sensitivity analysis of the comparison of hospital stay between the PECD group and the ACDF group. [file 13018_2022_3365_MOESM1_ESM.docx]

Figure S1. Excellent treatment results after PECD for cervical disc herniation

Figure S2. Good treatment results after PECD for cervical disc herniation

Figure S3. VAS scores at lastly follow-up after PECD for cervical disc herniation

Figure S4. Comparison of hospital stay between PECD group and ACDF group

Figure S5. Sensitivity analysis of excellent treatment results after PECD for cervical disc herniation

Figure S6. Sensitivity analysis of good treatment results after PECD for cervical disc herniation

Figure S7. Sensitivity analysis of VAS scores at lastly follow-up after PECD for cervical disc herniation

Figure S8. Sensitivity analysis of the comparison of hospital stay between PECD group and ACDF group
